# Real-World Effectiveness, Corticosteroid-Sparing Effects, and Exploratory Immunological Correlates of Omalizumab Add-On Therapy in Hospitalized Patients with Acute Urticaria

**DOI:** 10.3390/biomedicines14081802

**Published:** 2026-08-11

**Authors:** Tingting Jiang, Zhiqiang Zhang, Li Wu, Jing Li, Ruzhi Zhang

**Affiliations:** Department of Dermatology, The Second Affiliated Hospital of Wannan Medical University, Wuhu 241000, China; jiangtt0206@163.com (T.J.); zhiqiangzhang1126@163.com (Z.Z.); wuli2316@163.com (L.W.); jing035152@163.com (J.L.)

**Keywords:** acute urticaria, omalizumab, glucocorticoids, IgE, corticosteroid-sparing therapy, predictive model

## Abstract

**Background/Objectives**: Hospitalized patients with acute urticaria often present with rapid symptom progression, severe pruritus, angioedema, and a high risk of short-term relapse. Systemic glucocorticoids provide rapid anti-inflammatory control but raise concerns about cumulative exposure and adverse effects. This real-world study evaluates whether adding omalizumab to systemic glucocorticoids improves efficacy, safety, and steroid-sparing outcomes and explores associated immunological correlates. **Methods**: This retrospective cohort study evaluated the real-world, off-label use of omalizumab as add-on therapy in hospitalized patients with acute urticaria treated between 2023 and 2026. We compared UAS7 scores, itch VAS, resolution times, the primary efficacy outcome of complete remission within 7 days, the key secondary efficacy outcome of recurrence within 14 days, corticosteroid exposure, adverse events, and immune–inflammatory markers. Propensity score matching (PSM) was used to reduce imbalances in measured baseline covariates, and a multivariable exploratory model with LASSO regression identified factors linked to short-term outcomes. **Results**: A total of 73 patients met the eligibility criteria and were included in the study, including 39 patients who received conventional glucocorticoid-centered therapy and 34 patients who received additional omalizumab. Measured baseline characteristics were generally comparable, although imbalances remained in sex and selected leukocyte measures. The combination group showed faster UAS7 and VAS declines from Day 1, with widening differences at Days 3, 7, and 14. Combination therapy shortened the time to wheal and angioedema resolution, achieved a 7-day complete remission rate of 82.9% vs. 53.3%, and reduced 14-day recurrence to 17.1% vs. 40.0%. Total corticosteroid exposure and tapering duration were significantly reduced, with lower incidences of overall adverse events, gastrointestinal discomfort, and hyperglycemia. The exploratory model identified elevated neutrophil counts as a risk factor for poorer outcomes, while combination therapy was associated with reduced risk (bootstrap-corrected AUC 0.812, Brier score 0.126). **Conclusions**: In hospitalized patients with acute urticaria, adding omalizumab to systemic glucocorticoids is associated with faster symptom relief, lower short-term recurrence, reduced steroid exposure, and a better short-term safety profile. This combination may benefit selected patients with severe disease and angioedema, though prospective validation is warranted.

## 1. Introduction

Urticaria is a mast cell-driven disorder characterized by wheals, pruritus, and angioedema. Disease lasting ≤6 weeks is classified as acute, whereas persistence beyond 6 weeks defines chronic urticaria, consistent with the updated international guideline [1]. Urticaria affects both children and adults, although chronic forms are generally less common in pediatric patients. In children, the reported prevalence of chronic urticaria ranges from 0.1% to 3%, while the prevalence of pediatric chronic spontaneous urticaria has traditionally been estimated at 0.1–0.3%, although it may be higher than previously recognized [2]. Specific triggers cannot be identified in more than half of pediatric chronic urticaria cases, and autoimmune mechanisms may contribute to disease development in a subset of patients [3]. In adults, chronic urticaria is a heterogeneous disorder that may present with wheals, angioedema, or both and may be classified as spontaneous or inducible [4]. Among older adults, the reported prevalence of chronic urticaria ranges from 0.2% to 2.8%; women are more frequently affected, and wheals without angioedema represent the predominant clinical presentation [5].

Beyond a transient skin eruption, urticaria involves immune activation, neural responses, and altered vascular permeability [6] and contributes substantially to healthcare use, work loss, and impaired quality of life [7,8]. Although acute urticaria is often self-limiting, recent systematic reviews have shown that the evidence supporting its pharmacological management remains limited and heterogeneous, particularly regarding the incremental benefit of systemic corticosteroids when added to antihistamines [9,10]. The available trials are generally small and provide insufficient evidence to define optimal escalation strategies for hospitalized patients with severe acute urticaria. Management must therefore balance rapid symptom control with treatment safety and judicious corticosteroid use.

This challenge is complicated by heterogeneous triggers and presentations. Infections, medications, foods, physical stimuli, and unexplained immune activation may provoke disease, with manifestations ranging from localized wheals to angioedema, gastrointestinal symptoms, chest tightness, or anaphylaxis-like features. Because acute urticaria and anaphylaxis may initially overlap, clinicians must assess both cutaneous and systemic risk [11]. Emergency department studies also show considerable variation in diagnostic and therapeutic pathways [12]. Second-generation H1 antihistamines remain the first-line treatment, while severe or refractory cases often receive short courses of systemic glucocorticoids [1,13]. However, the limited evidence supporting treatment intensification highlights the need for therapies that achieve rapid control without unnecessarily increasing corticosteroid exposure [12,13].

From a pathophysiological perspective, urticaria results primarily from mast-cell degranulation triggered by immunological or non-immunological mechanisms, leading to transient wheals and/or angioedema [14]. Mast cells link immune signaling with vascular responses [15], while IgE–FcεRI signaling may lower the activation threshold of effector cells [16]. Basophils, eosinophils, neutrophils, and complement also contribute: basophils connect peripheral immune activity with skin reactions [17], and allergic, innate, and neuroimmune pathways may act jointly [18]. BTK signaling, FcεRI-mediated activation, and downstream inflammatory amplification further support acute symptom development [19,20]. FcεRI expression may reflect immunological activity [21], and upstream pathway inhibition may suppress mast-cell reactivity at its source [22]. Accordingly, disease activity may be better characterized by integrating symptom scores, IgE, inflammatory markers, and white-cell profiles than by any single measure.

Systemic glucocorticoids rapidly suppress inflammatory transcription, vascular leakage, wheals, and edema but act primarily downstream without neutralizing free IgE or modifying FcεRI activation. Short-term prednisolone may accelerate resolution in selected patients [23], whereas a randomized trial found no additional benefit of prednisone plus levocetirizine over levocetirizine alone in acute urticaria without angioedema [24]. Recent systematic reviews and meta-analyses have similarly reported inconsistent findings and limited certainty regarding the additional benefit of systemic corticosteroids in acute urticaria [11,12,25]. Accordingly, systemic corticosteroids should be viewed as a selective, short-term treatment rather than an established strategy for routine intensification. Even short courses have been associated with infection, venous thrombosis, fractures, endocrine disturbances, and glucose metabolism abnormalities [26,27,28,29,30], highlighting the need for corticosteroid-sparing strategies.

Omalizumab offers a complementary approach by binding free IgE, preventing its interaction with FcεRI, and reducing FcεRI expression on mast cells and basophils [31]. Translational studies confirm reduced basophil FcεRI expression and altered skin mast-cell function [32], while its inability to cross-link cell-surface IgE supports a favorable safety mechanism [33]. Basophil-related parameters, including peripheral basophil counts, FcεRI expression, cellular responsiveness or activation, and cytokine release, have therefore been proposed as candidate biomarkers for assessing or predicting the clinical response to omalizumab. However, their practical clinical utility remains investigational and has not been established in acute urticaria [34]. Omalizumab is licensed for allergic asthma and chronic spontaneous urticaria, among other indications, depending on the jurisdiction, but it is not approved for acute urticaria; therefore, its use for the patients evaluated in the present study was off-label [35,36]. In chronic spontaneous urticaria, randomized trials show reduced pruritus and wheal burden with good tolerability [37,38,39], including across different background treatments [40]. Meta-analyses further confirm its efficacy and safety [41,42,43,44]. However, whether these findings extend to hospitalized acute urticaria, particularly for rapid control, corticosteroid reduction, and early recurrence prevention, remains uncertain. Current evidence does not establish omalizumab as routine therapy for acute urticaria, and its use in this setting remains off-label. This evidence gap provides the rationale for evaluating omalizumab as a selective add-on strategy in a hospitalized real-world cohort while interpreting the findings as exploratory.

This distinction is clinically important because chronic disease emphasizes long-term control and quality of life, whereas acute severe disease requires relief within hours to days. UAS7 provides a validated assessment of wheals and itch [45,46], but evaluation should also include disease control and patient-reported outcomes [47]. Since Finlay and Khan developed the DLQI, studies have linked urticaria activity and inflammatory biomarkers to dermatology-related quality of life [48,49], while anxiety, depression, and sleep disturbance have been shown to further influence disease burden [50,51]. Biomarkers may reflect the immunopathological processes underlying urticaria and contribute to the assessment of disease activity, prognosis, treatment response, and risk stratification [52,53]. Prediction models should therefore report variable selection, discrimination, calibration, and clinical net benefit according to updated reporting guidance applicable to both regression and machine learning models [54,55]. Decision curve analysis evaluates usefulness across risk thresholds [56], and a good AUC alone is insufficient without agreement between predicted and observed risk [57].

Against this background, we compared hospitalized patients receiving systemic glucocorticoids with those receiving omalizumab plus glucocorticoids. We evaluated longitudinal UAS7 and itch VAS scores, wheal and angioedema resolution, remission, recurrence, corticosteroid exposure, safety, and immune–inflammatory markers. Correlation analysis, hierarchical clustering, multivariable regression, ROC analysis, and calibration were used to explore potential factors associated with short-term outcomes. We hypothesized that upstream IgE blockade combined with downstream inflammatory suppression might be associated with faster relief, lower recurrence, reduced corticosteroid exposure, and improved safety.

## 2. Materials and Methods

### 2.1. Study Design and Participants

This retrospective cohort study was conducted at a single center, enrolling hospitalized patients with acute urticaria who were admitted and treated between 2023 and 2026, had completed treatment, and had traceable medical records. Both pediatric and adult patients were eligible for inclusion; pediatric patients were defined as those aged <18 years and adults as those aged ≥18 years. No age-based exclusion criterion was applied. Acute urticaria was defined as the sudden onset of wheals and pruritus, with or without angioedema, and a total disease duration not exceeding six weeks.

Eligibility was assessed according to the following predefined inclusion and exclusion criteria. Inclusion criteria were: (1) pediatric or adult patients with a clinical diagnosis of acute urticaria; (2) documented in-hospital treatment regimen; (3) availability of baseline symptom scores or retrievable symptom descriptions; and (4) completed key laboratory tests. Exclusion criteria included: disease course consistent with chronic urticaria; primary diagnosis of drug eruption, vasculitic urticaria, hereditary angioedema, or other non-IgE-mediated urticarial disorders; missing key outcome data; or presence of severe infections, malignancies, or immunodeficiency.

After application of the inclusion and exclusion criteria, eligible patients were classified into the corticosteroid group or the combination therapy group according to whether they received subcutaneous omalizumab in addition to standard therapy. To mitigate potential selection bias, we applied propensity score matching (PSM) using a 1:1 nearest-neighbor algorithm with a caliper of 0.2. However, PSM was necessarily limited to measured covariates available in the medical records and could not account for undocumented clinical judgment, corticosteroid contraindications or intolerance, affordability, insurance coverage, drug availability, or other factors that may have influenced treatment selection. The matched cohort was used for sensitivity analyses to validate the robustness of primary findings.

This study adhered to the ethical principles set forth in the Declaration of Helsinki [58]. The study protocol was reviewed and approved by the Human Research Ethics Committee of the Second Affiliated Hospital of Wannan Medical University, with approval number WYEFYL2026071 and approval date 11 July 2026. Before patients’ clinical data were included in this study, the researchers explained the purpose of the study, its content, how the clinical data would be used, and the relevant privacy protection measures to all study participants and obtained their informed consent. This research consent was distinct from the clinical consent for off-label omalizumab treatment. Before omalizumab administration, patients or their legal guardians were informed that its use for acute urticaria was off-label and were counseled regarding the clinical rationale, limited evidence base, potential benefits and risks, available alternatives, treatment costs, and possible uncertainty in insurance or reimbursement coverage. Written informed consent specific to off-label omalizumab use was obtained.

### 2.2. Treatment Protocols and Exposure Assessment

Patients in the corticosteroid group received systemic glucocorticoids (methylprednisolone or dexamethasone) combined with second-generation H1 antihistamines, with or without H2-receptor antagonists or leukotriene receptor antagonists as needed. The combination therapy group received subcutaneous omalizumab (at recorded doses of 150 mg, 300 mg, or 450 mg) in addition to the above regimen. Because omalizumab is not approved for the treatment of acute urticaria, its administration in the combination therapy group constituted off-label use. Treatment decisions were made individually by the treating physicians after considering disease severity, angioedema, response to conventional therapy, anticipated benefits and risks, and patient preferences. There was no prespecified institutional algorithm or uniform clinical threshold for adding omalizumab. The retrospective medical records did not systematically document whether corticosteroid contraindications or intolerance, treatment cost, insurance or reimbursement coverage, or immediate drug availability contributed to the treatment decision for each patient.

To ensure dose comparability, all systemic glucocorticoid doses were converted to methylprednisolone equivalents using standard conversion factors. Cumulative corticosteroid exposure for each patient was calculated as the sum of each administered dose multiplied by its respective conversion factor. The average daily exposure was derived by dividing the cumulative exposure by the total number of days of systemic corticosteroid use. The corticosteroid tapering period was defined as the interval from treatment initiation to the first dose reduction or discontinuation; for patients who did not complete tapering during hospitalization, the period was truncated at the last traceable medication record.

### 2.3. Outcome Measures

The primary efficacy outcome was complete remission within 7 days, defined as resolution of wheals, substantial itch relief, absence of new angioedema episodes, and no need for additional systemic corticosteroid rescue therapy. Recurrence within 14 days was designated as a key secondary efficacy outcome. Other secondary efficacy outcomes included: dynamic changes in UAS7 and pruritus visual analog scale (VAS) scores over the treatment course; time to wheal resolution; time to angioedema resolution; complete remission rate within 7 days; recurrence rate within 14 days; and total length of hospital stay. The symptom reduction rate at each time point was calculated as the percentage decrease from baseline.

Safety outcomes encompassed overall adverse events, gastrointestinal discomfort, hyperglycemia, insomnia or anxiety, blood pressure fluctuations, and serious adverse events. Recurrence was defined as the reappearance of urticaria, pruritus, or angioedema after remission, necessitating additional antihistamines, systemic corticosteroids, or an unscheduled return visit.

### 2.4. Laboratory Parameters and Derived Inflammatory Indices

Laboratory measurements collected at admission included white blood cell (WBC) count, absolute neutrophil count (NEU), absolute eosinophil count (EOS), lymphocyte count (LYM), platelet count (PLT), C-reactive protein (CRP), erythrocyte sedimentation rate (ESR), and total IgE. To enhance the inflammatory characterization, we derived the neutrophil-to-lymphocyte ratio (NLR = NEU/LYM), platelet-to-lymphocyte ratio (PLR = PLT/LYM), and systemic immune–inflammation index (SII = PLT × NEU / LYM). Given the right-skewed distributions of total IgE and CRP, these variables were log-transformed [ln(value + 1)] for correlation analyses and model building.

### 2.5. Statistical Analysis

Continuous variables were presented as means ± SD or medians (IQRs) and categorical variables as frequencies (percentages), with between-group comparisons performed using the *t*-test, Mann–Whitney U test, or chi-square test as appropriate. Baseline demographic, clinical, and laboratory characteristics were summarized in a dedicated table for the overall cohort and by treatment group. For variables with missing observations, the available denominator was reported. The omalizumab dose was summarized descriptively according to recorded administrations. Because dose assignment was nonrandom and the 150, 300, and 450 mg groups were small and unevenly distributed, no formal dose–response analysis was prespecified. Consequently, associations between omalizumab dose and the speed of symptom improvement were not subjected to confirmatory statistical testing. Because multiple secondary efficacy, safety, laboratory, correlation, and model analyses were performed, no formal adjustment for multiple testing was applied. The corresponding *p*-values were therefore interpreted as nominal, and these analyses were regarded as exploratory and hypothesis-generating [59].

For repeated measures of UAS7 and itch VAS, linear mixed-effects models with a random patient intercept were applied, incorporating treatment group, time, and their interaction as fixed effects. A significant interaction indicated that combination therapy altered the overall trajectory of symptom improvement. Multivariate logistic regression was used for binary outcomes (e.g., 7-day complete remission), adjusting for baseline covariates including treatment assignment, age, sex, Body Mass Index (BMI), IgE, UAS7, CRP, NEU, EOS, comorbidities, and cumulative corticosteroid dose. To construct an exploratory predictive model for short-term outcomes while avoiding overfitting in this modest sample, we employed LASSO regression for variable selection, prioritizing clinical interpretability alongside coefficient stability. Model discrimination was assessed by the area under the ROC curve (AUC) and calibration by calibration plots with the Brier score; clinical utility was further explored via decision curve analysis. All tests were two-sided, with significance set at *p* < 0.05, and analyses were conducted using R software (version 4.2, R Foundation for Statistical Computing, Vienna, Austria).

## 3. Results

### 3.1. Baseline Demographic, Clinical, and Laboratory Characteristics and IgE Immunophenotypes

A total of 73 patients met the eligibility criteria and were included in the final analysis, including 39 patients in the corticosteroid group and 34 patients in the combination therapy group. The study population included both pediatric and adult patients, with an overall age range of 8–86 years. Detailed demographic, clinical, and laboratory characteristics of the overall cohort and the two treatment groups are presented in Table 1. The two groups were generally comparable with respect to age, body weight, BMI, precipitating factors, alcohol consumption history, comorbidities, and most laboratory parameters.

**Figure 1:**
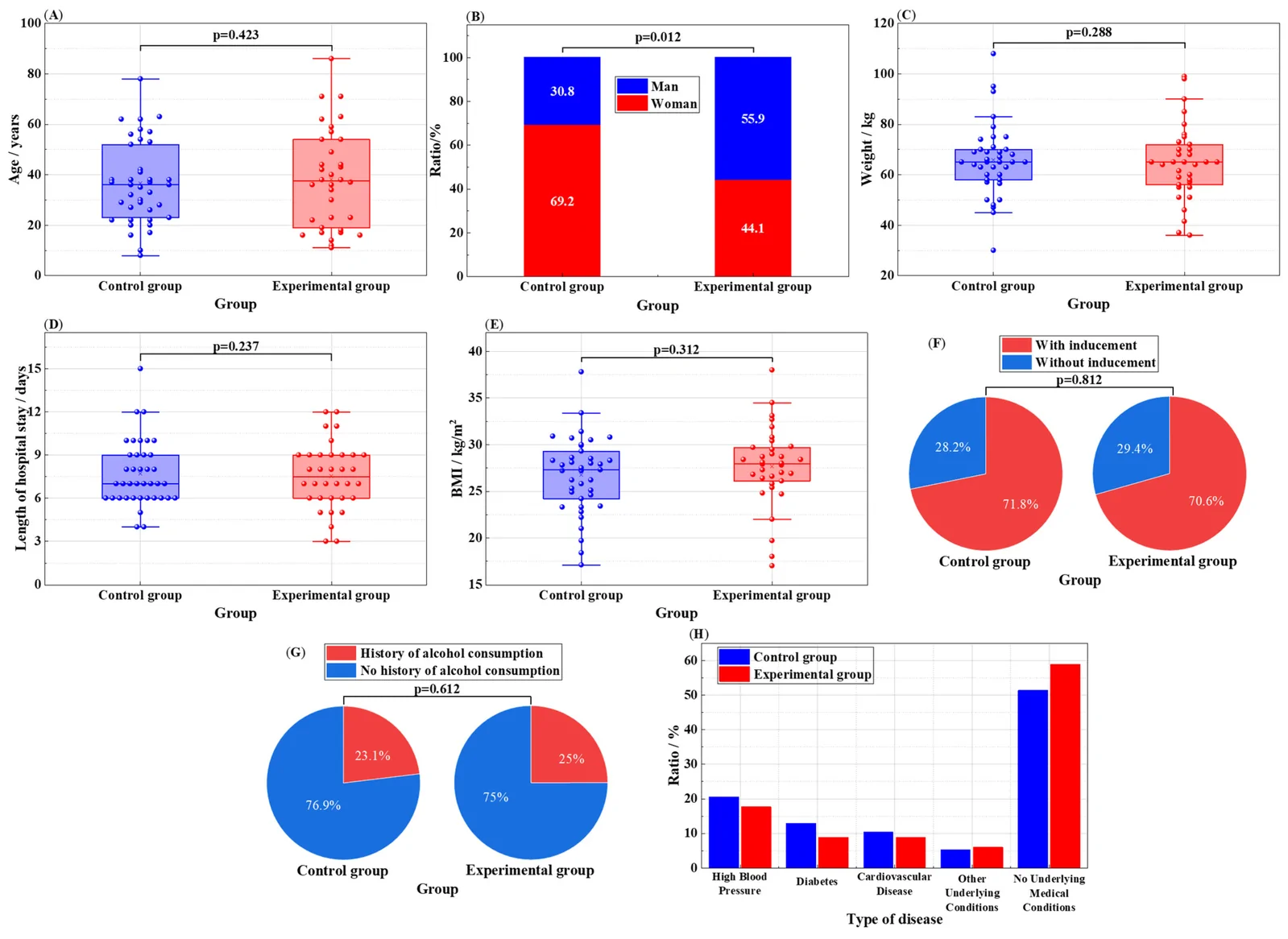
Baseline demographic and clinical characteristics: (**A**) age distribution; (**B**) sex distribution; (**C**) body weight distribution; (**D**) length of hospital stay; (**E**) BMI distribution; (**F**) prevalence of precipitating factors; (**G**) prevalence of alcohol use; (**H**) prevalence of comorbidities.

Before evaluating combination-therapy efficacy, demographic and clinical characteristics at admission were compared between the groups to assess the potential influence of baseline imbalances (Figure 1). The comparison included age, sex, body weight, length of hospital stay, BMI, history of precipitating factors, alcohol consumption, and comorbidities. Age distributions and medians were similar between groups (*p* = 0.423), indicating that the combination therapy group was not systematically younger or older. Sex distribution differed significantly: 69.2% of patients in the corticosteroid group were female and 30.8% were male, compared with 44.1% and 55.9%, respectively, in the combination therapy group (*p* = 0.012); the potential effect of sex was therefore considered in subsequent analyses. Median body weight was similar between groups, although variability was slightly greater in the combination therapy group (*p* = 0.288), and BMI was also comparable (*p* = 0.312). Length of hospital stay was similar between groups (*p* = 0.237). The median was 7.0 days in the corticosteroid group and 7.5 days in the combination therapy group. Histories of precipitating factors were present in 71.8% of the corticosteroid group and 70.6% of the combination group (*p* = 0.812). Alcohol consumption histories were present in 23.1% and 25.0%, respectively (*p* = 0.612). The distribution of hypertension, diabetes, cardiovascular disease, other comorbidities, and no comorbidity was also similar. Overall, except for sex distribution, the groups were comparable with respect to age, weight, BMI, length of hospital stay, histories of precipitating factors and alcohol consumption, and comorbidities.

Baseline results indicate that the groups were generally well balanced with respect to age, weight, BMI, length of hospital stay, histories of precipitating factors and alcohol consumption, and comorbidities. The sex imbalance was accounted for in model interpretation. Inflammatory and complete blood count parameters were subsequently compared to determine whether the groups differed in immune–inflammatory phenotype.

**Figure 2:**
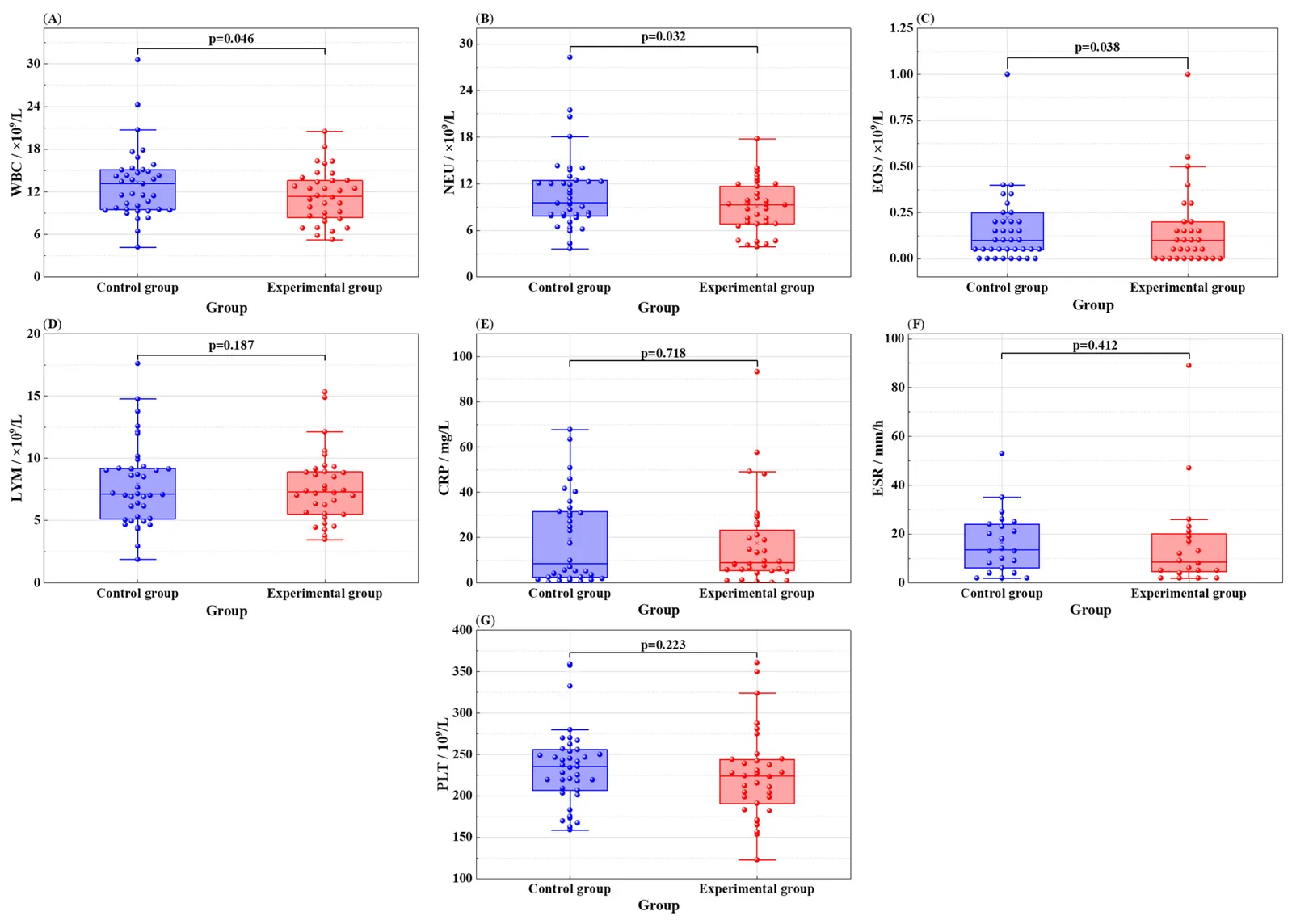
Distribution of inflammatory and complete blood count parameters: (**A**) white blood cell count; (**B**) neutrophil count; (**C**) eosinophil count; (**D**) lymphocyte count; (**E**) CRP level; (**F**) ESR level; (**G**) platelet count.

After confirming general comparability of demographic and clinical characteristics, we compared immune and inflammatory markers at admission to determine whether inflammatory states differed before treatment (Figure 2). White blood cell count differed significantly between groups (*p* = 0.046), with slightly higher overall values in the corticosteroid group, suggesting that the peripheral inflammatory response at admission was not entirely equivalent. Neutrophil counts also differed (*p* = 0.032), with slightly higher medians and upper quartiles in the corticosteroid group, suggesting a difference in innate inflammatory burden. Eosinophil counts likewise differed (*p* = 0.038). Although both groups showed many low values and a few high values, their overall distributions were different, suggesting that an allergy-related inflammatory phenotype may contribute to the clinical heterogeneity of acute urticaria. In contrast, lymphocyte count, CRP, ESR, and platelet count did not differ significantly. Lymphocyte distributions were similar (*p* = 0.187). CRP was widely dispersed in both groups, with isolated high values but no between-group difference (*p* = 0.718). Similarly, some patients had elevated ESR, but the overall difference was not significant (*p* = 0.412). Platelet medians and interquartile ranges were also similar (*p* = 0.223). Thus, admission differences were concentrated in white blood cell, neutrophil, and eosinophil counts, whereas CRP, ESR, lymphocyte count, and platelet count did not differ significantly.

These findings indicate that analyses of omalizumab combination therapy should account for pretreatment differences in peripheral inflammatory cells, particularly the inflammatory status reflected by neutrophils and eosinophils.

**Figure 3:**
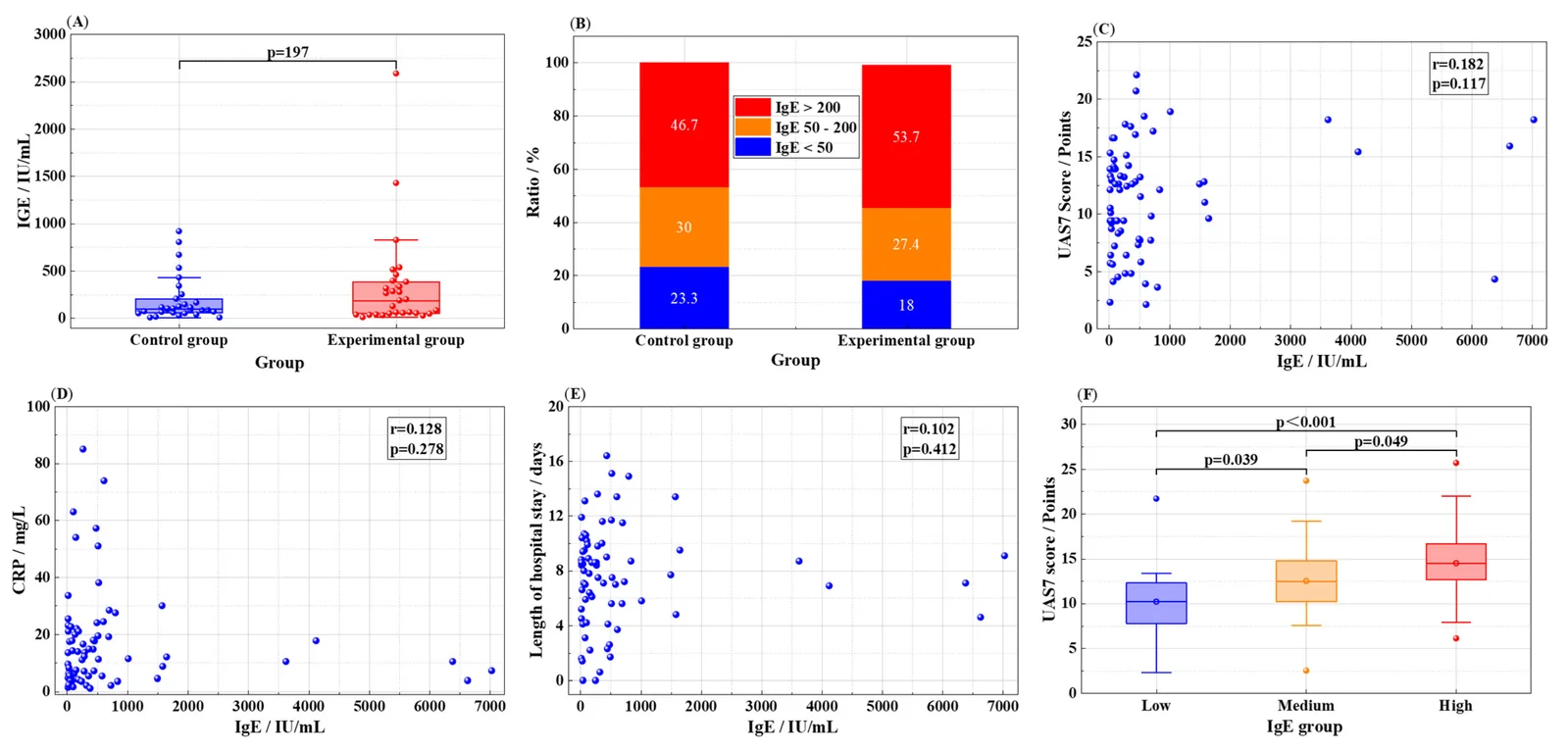
Analysis of IgE levels and immunological parameters: (**A**) total IgE distributions in the two groups; (**B**) proportions of patients in each IgE subgroup; (**C**) correlation between total IgE and UAS7; (**D**) correlation between total IgE and CRP; (**E**) correlation between total IgE and length of hospital stay; (**F**) UAS7 distributions across IgE subgroups.

Several granulocyte-related inflammatory variables differed between groups, whereas omalizumab primarily targets IgE. To determine whether IgE burden was associated with symptom activity, systemic inflammation, and hospital outcomes, we analyzed both continuous total IgE values and IgE strata (Figure 3). Total IgE distribution did not differ significantly between groups (*p* = 0.197). Although the combination therapy group included higher IgE outliers, the two boxplots overlapped substantially, indicating that this group was not composed solely of patients with markedly high or low IgE. After stratification, low-, moderate-, and high-IgE patients were represented in both groups. In the corticosteroid group, 23.3%, 30.0%, and 46.7% had low, moderate, and high IgE, respectively; the corresponding proportions in the combination group were 18.0%, 27.4%, and 53.7%. Although the proportion with high IgE was slightly greater in the combination group, the overall distribution showed no meaningful imbalance. Continuous total IgE had a weak, nonsignificant positive correlation with UAS7 (r = 0.182, *p* = 0.117), a weak correlation with CRP (r = 0.128, *p* = 0.278), and a weak correlation with length of hospital stay (r = 0.102, *p* = 0.412). Thus, continuous IgE alone had limited ability to explain symptom severity, systemic inflammation, or length of stay. In contrast, IgE strata showed a clearer UAS7 gradient: UAS7 was lowest in the low-IgE group, higher in the moderate-IgE group than in the low-IgE group (*p* = 0.039), and still higher in the high-IgE group than in the moderate-IgE group (*p* = 0.049); the high- versus low-IgE contrast was most pronounced (*p* < 0.001).

Continuous total IgE levels showed limited linear associations with individual outcomes, whereas IgE stratification revealed differences in symptom activity. Total IgE distribution and IgE strata were similar between the groups, making it unlikely that baseline IgE imbalance materially influenced the subsequent efficacy comparison. A clear gradient between IgE strata and UAS7 was nevertheless observed, suggesting that higher IgE burden may be associated with greater disease activity. These findings provide a mechanistic rationale for further analysis of omalizumab combination therapy and suggest that modulation of IgE-related pathways may contribute to symptom remission.

### 3.2. Dynamic Symptom Management and Short-Term Efficacy

**Figure 4:**
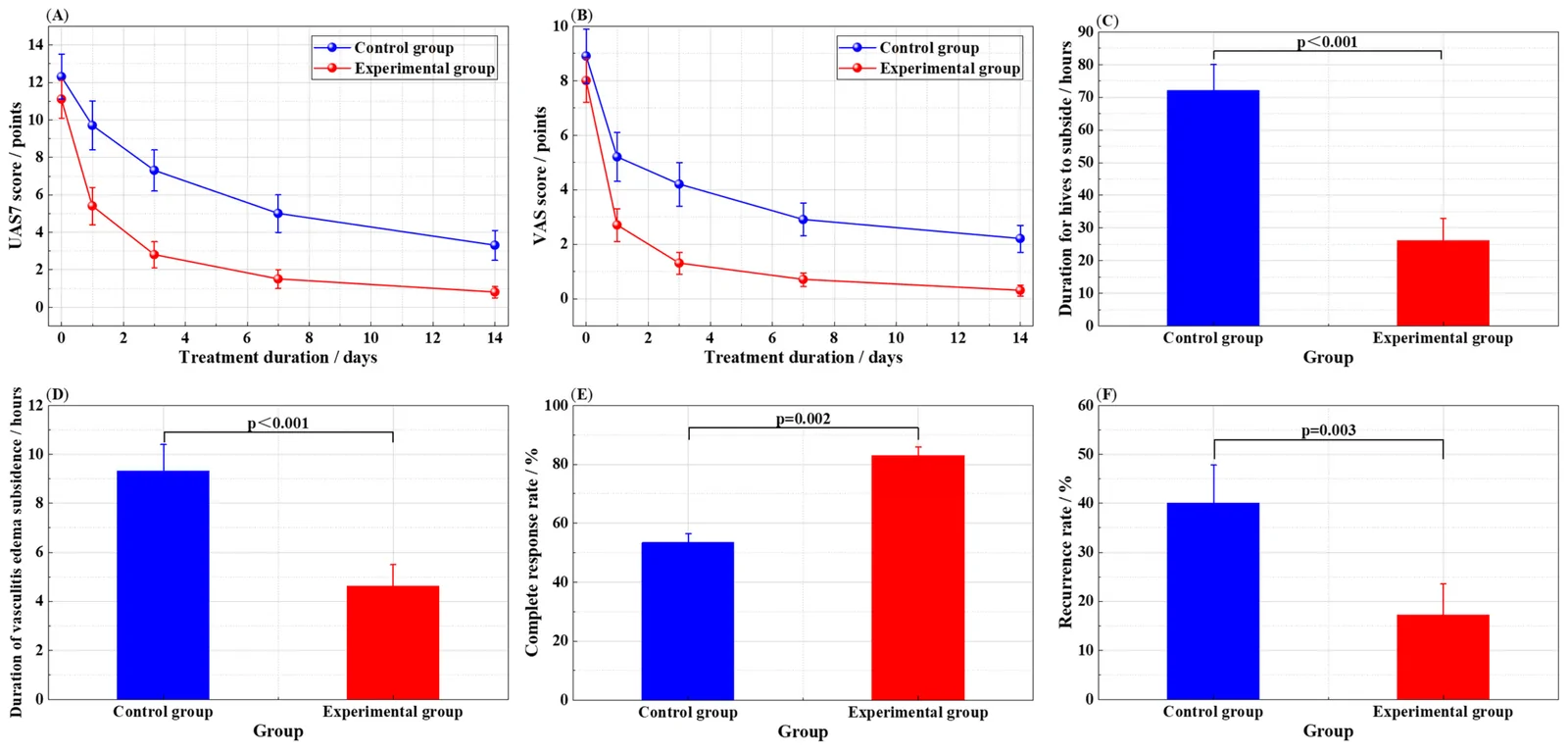
Urticaria activity and symptom scores: (**A**) changes in UAS7 over time; (**B**) changes in itch VAS over time; (**C**) time to resolution of wheals; (**D**) time to resolution of angioedema; (**E**) complete remission rate at 7 days; (**F**) recurrence rate at 14 days. Data are presented as mean ± standard deviation (SD), and the error bars indicate the SD of the measurements.

After the baseline and immunophenotyping analyses, we compared the effects of the two regimens on symptom relief and short-term outcomes (Figure 4). Pretreatment UAS7 scores were similar, indicating high disease activity in both groups. Scores decreased over time in both groups, but the decline was faster in the combination therapy group. By Day 1, UAS7 was already significantly lower in the combination therapy group, and the difference widened on Day 3. On Days 7 and 14, its scores remained lower, indicating more rapid reduction in disease activity and better symptom control during follow-up. Itch VAS followed a similar pattern. Baseline itch severity was comparable, but the combination therapy group experienced a rapid decline after treatment: VAS was lower by Day 1, fell further after Day 3, and remained low on Days 7 and 14. These early benefits were first evident in pruritus relief and were consistent with the overall reduction in urticaria activity. The time to resolution of wheals and angioedema was significantly shorter in the combination therapy group than in the corticosteroid group (both *p* < 0.001), indicating that combination therapy accelerated resolution of both visible skin lesions and deep edema. For the primary efficacy outcome, the Day 7 complete remission rate was 82.9% in the combination therapy group versus 53.3% in the corticosteroid group (*p* = 0.002). For the key secondary efficacy outcome, the 14-day recurrence rate was 17.1% versus 40.0%, respectively (*p* = 0.003). Overall, combination therapy was associated with earlier itch relief, reduced disease activity, faster resolution of lesions and edema, higher 7-day complete remission, and lower 14-day recurrence, suggesting faster onset and more stable short-term control than corticosteroid-based treatment alone.

To clarify the clinical significance of efficacy differences beyond *p*-values, the primary and key secondary efficacy outcomes were converted into absolute and relative effect measures (Table 2). Combination therapy increased the 7-day complete remission rate by 29.6% (RR = 1.56), corresponding to approximately one additional patient achieving complete remission within 7 days for every four patients treated. The 14-day recurrence rate decreased by 22.9%, a relative reduction of 57.3%, corresponding to approximately one short-term recurrence prevented for every five patients treated. Resolution of both wheals and angioedema was significantly faster in the combination therapy group, and early declines in UAS7 and itch VAS indicate that treatment differences emerged as early as Day 1.

Symptom-dynamics analyses show that the advantage of combination therapy is not confined to a single statistical endpoint; rather, it extends from early itch relief to wheal and edema resolution, complete remission, and recurrence prevention. The next question was whether these efficacy gains were achieved at the cost of greater drug exposure; drug-use patterns were therefore analyzed further.

### 3.3. Drug Exposure, Safety, and Follow-Up Outcomes

**Figure 5:**
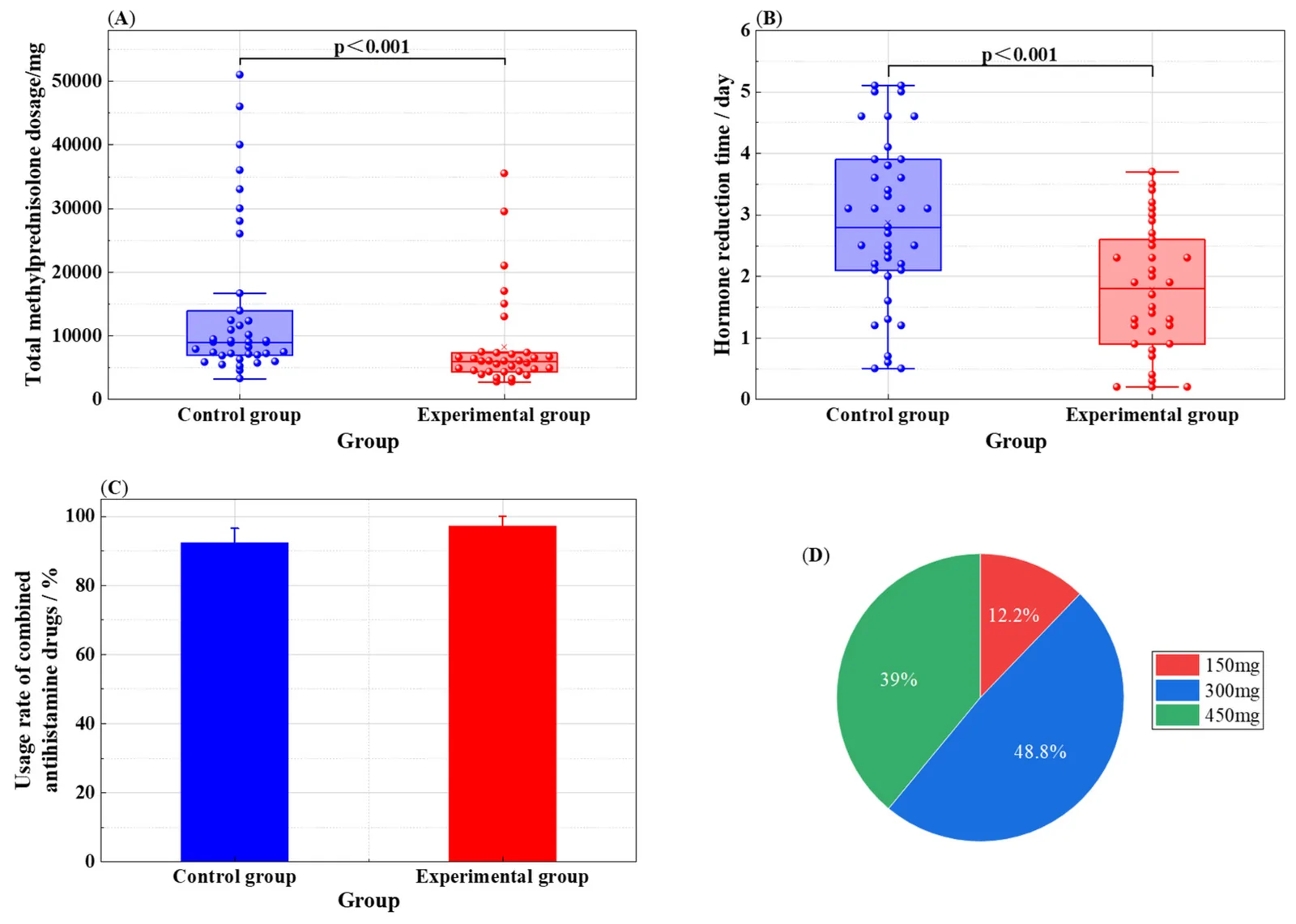
Treatment regimens and medication use: (**A**) total methylprednisolone-equivalent corticosteroid dose; (**B**) duration of corticosteroid tapering; (**C**) rate of concomitant antihistamine use; (**D**) distribution of omalizumab injection doses.

Whether improved efficacy is achieved at the cost of greater drug exposure is central to assessing the clinical value of the combination regimen. We therefore compared total corticosteroid dose, time to corticosteroid tapering, concomitant antihistamine use, and omalizumab dose distribution (Figure 5). The total methylprednisolone-equivalent dose was significantly lower in the combination therapy group than in the corticosteroid group (*p* < 0.001), indicating a clear corticosteroid-sparing effect. Time to corticosteroid tapering was also shorter in the combination therapy group (*p* < 0.001), indicating that systemic corticosteroids could be tapered earlier once symptom control improved. Concomitant antihistamine use was high in both groups, at approximately 93% in the corticosteroid group and nearly 98% in the combination group, indicating that baseline antiallergic treatment was adequate in both groups. Thus, superior efficacy in the combination group cannot be attributed simply to different antihistamine use. Omalizumab doses were concentrated at 300 mg and 450 mg, accounting for 48.8% and 39.0% of administrations, respectively, while 150 mg accounted for the remaining 12.2%. These percentages represent recorded administrations rather than proportions of individual patients. The omalizumab dose was not matched to baseline total IgE or body weight. Because the dose groups were small, unevenly distributed, and selected nonrandomly, no reliable association between dose and the speed of UAS7 reduction, itch relief, or wheal and angioedema resolution could be established.

Drug exposure analyses indicate that the combination regimen did not achieve better efficacy through greater reliance on conventional antihistamines. Instead, it improved symptom control while reducing total corticosteroid dose and shortening the tapering period. Because lower systemic corticosteroid exposure may improve safety, adverse reactions and recurrence were subsequently assessed.

**Figure 6:**
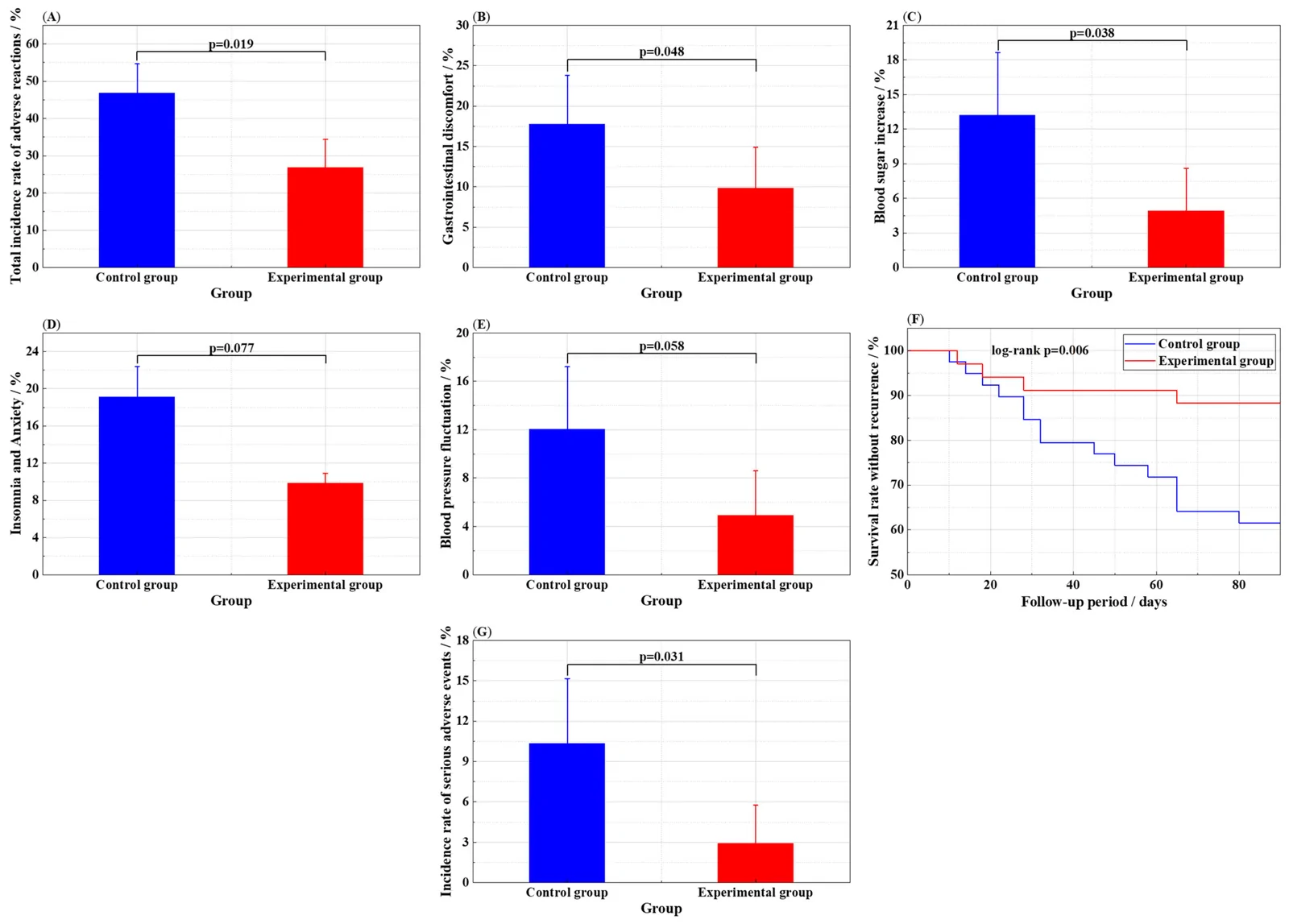
Safety and short-term follow-up outcomes: (**A**) overall incidence of adverse events; (**B**) incidence of gastrointestinal discomfort; (**C**) incidence of elevated blood glucose; (**D**) incidence of insomnia and anxiety; (**E**) incidence of blood pressure fluctuations; (**F**) recurrence-free survival curve; (**G**) incidence of serious adverse events. Data are presented as mean ± standard deviation (SD), and the error bars indicate the SD of the measurements.

After confirming the corticosteroid-sparing effect of combination therapy, we evaluated safety. To determine whether adding omalizumab increased risk and whether corticosteroid-related adverse reactions decreased with lower corticosteroid exposure, we compared overall adverse reactions, gastrointestinal discomfort, hyperglycemia, insomnia and anxiety, blood pressure fluctuations, recurrence-free survival, and serious adverse events (Figure 6). Overall adverse events were less common in the combination therapy group than in the corticosteroid group (*p* = 0.019), indicating that improved efficacy was not accompanied by a higher overall safety burden. Gastrointestinal discomfort (*p* = 0.048) and elevated blood glucose (*p* = 0.038) were also less frequent in the combination group, suggesting that corticosteroid-related adverse reactions may decrease as exposure declines. Insomnia or anxiety (*p* = 0.077) and blood pressure fluctuations (*p* = 0.058) were numerically less frequent in the combination group, although the differences were not statistically significant, indicating a downward trend. During follow-up, recurrence-free survival curves diverged, with the combination group showing higher overall recurrence-free survival, consistent with the lower recurrence rate. Serious adverse events were also less frequent in the combination group, although the difference was not statistically significant (*p* = 0.031), providing no evidence that combination therapy increased serious safety risk. Overall, combination therapy improved efficacy and reduced recurrence while being associated with fewer overall and certain corticosteroid-related adverse reactions, supporting a favorable short-term risk–benefit profile.

To facilitate interpretation of safety differences for clinical decision-making, absolute risk differences and relative risk changes were also calculated (Table 3). In the combination group, overall adverse events decreased by 19.9% in absolute terms and 42.6% in relative terms, corresponding to approximately one adverse event prevented for every six patients treated. Gastrointestinal discomfort, hyperglycemia, and serious adverse events each decreased; hyperglycemia showed a 62.9% relative reduction, which may be particularly important for patients at risk of glucose metabolism abnormalities. Although insomnia, anxiety, and blood pressure fluctuations showed mainly downward trends, the direction of change was consistent with lower corticosteroid exposure.

Taken together, safety and follow-up results suggest that the clinical value of combination therapy derives not only from more rapid remission but also from lower corticosteroid exposure and more stable short-term recurrence-free status. To identify factors associated with outcomes, we next developed a multivariable prediction model.

### 3.4. Multivariate Predictive Models and Correlation Cluster Analysis

**Figure 7:**
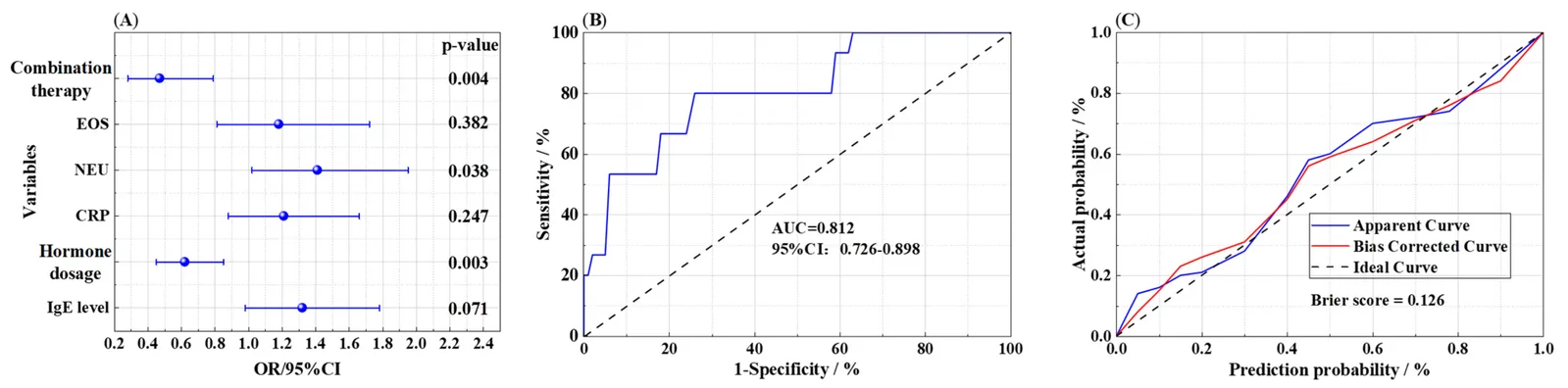
Multivariable analysis and prediction model: (**A**) multivariable logistic regression forest plot; (**B**) ROC curve; (**C**) calibration curve.

The preceding results indicated advantages of combination therapy in both efficacy and safety; however, the factors most strongly associated with short-term disease control required further evaluation. We therefore established a multivariable logistic regression model and assessed predictive performance with ROC and calibration curves (Figure 7). The forest plot showed that combination therapy was associated with better short-term disease control (OR approximately 0.47, 95% CI 0.28–0.79; *p* = 0.004), suggesting an independent protective association. Corticosteroid dose was also significantly associated with outcomes (OR approximately 0.62, 95% CI approximately 0.45–0.85; *p* = 0.003), consistent with the corticosteroid-sparing effect observed with combination therapy. Elevated NEU was significantly associated with outcome (OR approximately 1.41, 95% CI approximately 1.02–1.95; *p* = 0.038), suggesting that neutrophilic inflammatory burden may influence short-term disease control. Elevated IgE showed a borderline association (OR approximately 1.32, 95% CI approximately 0.98–1.78; *p* = 0.071), indicating possible predictive value that requires confirmation in a larger sample. CRP and EOS were not statistically significant (*p* = 0.247 and *p* = 0.382, respectively), indicating that single conventional inflammatory markers do not fully explain short-term outcomes. The model AUC was 0.812 (95% CI 0.726–0.898), indicating good discrimination. The apparent and bias-corrected calibration curves were generally close to the ideal curve, and the Brier score was 0.126, indicating good agreement between predicted and observed probabilities.

To move beyond the forest plot, we further summarized the clinical implications of key variables. Table 4 describes their direction of association, statistical significance, and possible interpretation. Combination therapy was the most consistently protective factor; elevated NEU indicated a greater risk of poor disease control; and IgE may reflect upstream allergic immune burden.

Practical value depends not only on the significance of individual variables but also on overall discrimination and calibration. Table 5 summarizes predictive model performance, including AUC, 95% CI, Brier score, and calibration curves. These findings indicate good internal perfformance but require validation in larger samples and external cohorts.

The predictive model suggests that NEU and IgE-related variables jointly influence short-term outcomes, supporting the presence of a recognizable immuno-inflammatory network in acute urticaria. To examine relationships among these variables at the network level, we generated correlation heatmaps and performed hierarchical cluster analysis.

**Figure 8:**
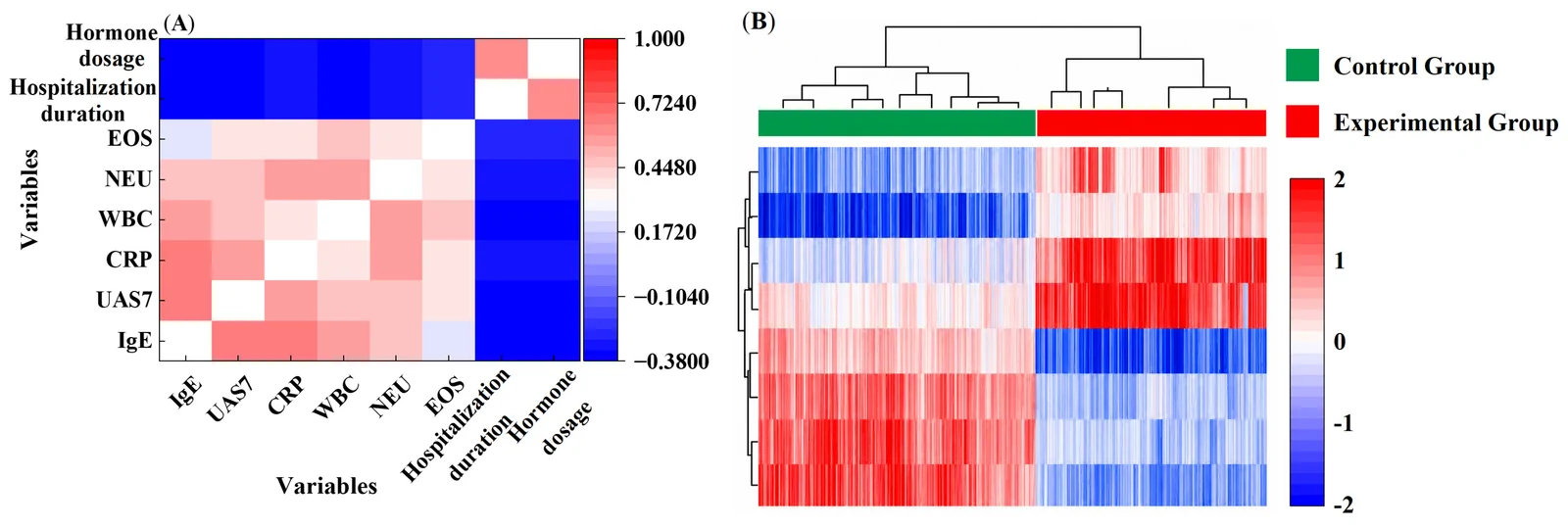
Correlation heatmap and cluster analysis: (**A**) correlations among IgE, UAS7, CRP, WBC, NEU, EOS, length of hospital stay, and corticosteroid dose; (**B**) hierarchical clustering of IgE, UAS7, CRP, NEU, EOS, corticosteroid dose, length of hospital stay, and WBC.

The prediction model identified IgE, NEU, and treatment exposure as variables associated with outcomes. To assess whether these variables form an interconnected immuno-inflammatory network, we generated a correlation heatmap and performed hierarchical clustering (Figure 8). The heatmap showed positive correlations of IgE with UAS7 and CRP. UAS7 was also positively correlated, to varying degrees, with CRP, WBC, NEU, and EOS, suggesting that symptom activity changes alongside immune burden and granulocytic inflammation rather than in isolation. WBC was strongly correlated with NEU, consistent with the complete blood count structure. Length of hospital stay and corticosteroid dose were positively correlated, suggesting a link between treatment intensity and the course of hospitalization; both were inversely related to IgE, UAS7, and inflammatory markers, possibly reflecting post-treatment reductions in inflammatory burden and clinical management. Hierarchical clustering separated patients into two major subgroups: one with lower IgE and inflammatory activity and the other with higher immune–inflammatory activity and greater treatment requirements. These findings suggest that acute urticaria may include identifiable immune–inflammatory subtypes.

Taken together, the results support a coherent conclusion. The 39 patients in the corticosteroid group and 34 patients in the combination therapy group were comparable on most baseline variables, and sex was accounted for as a potential confounder in the model. In this dataset, WBC, NEU, EOS, and IgE were more informative indicators of acute urticaria activity than CRP and ESR. The combination therapy group showed greater reductions in UAS7 and itch VAS from Day 1, with the advantage widening on Days 3, 7, and 14. It also had shorter times to resolution of wheals and angioedema, higher 7-day complete remission, and lower 14-day recurrence. Combination therapy reduced total corticosteroid exposure and tapering time and was associated with fewer corticosteroid-related adverse reactions. Modeling and clustering results further suggest that these benefits may reflect synergistic upstream IgE blockade, modulation of granulocyte-related inflammation, and corticosteroid-sparing effects.

## 4. Discussion

Most baseline variables were comparable between the corticosteroid group (*n* = 39) and the combination group (*n* = 34), with sex imbalance adjusted in the model. Against this background, omalizumab plus systemic glucocorticoids was associated with faster and more stable short-term improvement in hospitalized patients with acute urticaria. UAS7 and itch VAS declined more rapidly from Day 1, wheals and angioedema resolved sooner, the 7-day complete remission rate increased from 53.3% to 82.9%, and the 14-day recurrence rate decreased from 40.0% to 17.1%. Thus, the benefit extended from early symptom relief to lesion resolution, remission, and recurrence prevention.

These findings should be interpreted in the context of current treatment evidence. Second-generation H1 antihistamines remain the standard therapy, while systemic glucocorticoids are generally reserved for severe or inadequately controlled disease [1,13]. However, evidence for corticosteroid intensification is inconsistent. Pollack et al. reported symptomatic benefit from short-term prednisone [60], whereas Barniol et al. found no added effect of prednisone plus levocetirizine in acute urticaria without angioedema [24]. Palungwachira et al. similarly observed no additional benefit from intravenous dexamethasone in uncomplicated acute urticaria [61]. Systematic reviews also indicate limited and heterogeneous evidence, with possible symptom improvement offset by increased adverse events [15,19]. Corticosteroids should therefore be viewed as a short-term, risk-stratified treatment rather than the sole determinant of disease control.

Unlike many previous outpatient or emergency department studies, the present study focused on hospitalized patients with a greater symptom burden and more frequent angioedema. Baseline UAS7 and itch VAS were similar between groups, while treatment curves diverged by Day 1. This early difference may reflect complementary mechanisms: omalizumab suppresses upstream IgE-mediated activation, whereas glucocorticoids rapidly inhibit downstream inflammatory amplification. The higher recurrence rate and greater cumulative corticosteroid exposure in the corticosteroid group further suggest that downstream suppression alone may not stabilize disease in all highly active cases, particularly when IgE-related effector-cell activation persists.

The IgE findings support this interpretation. Total IgE and IgE strata were similar between groups, and continuous IgE correlated only weakly with UAS7, CRP, and hospital stay. However, UAS7 increased progressively across low-, moderate-, and high-IgE strata. Total IgE may therefore reflect immunological background rather than serve as a standalone severity marker. Importantly, baseline total IgE was not used to determine the omalizumab dose in this cohort. This differs from the IgE- and body-weight-based dosing algorithm used for allergic asthma and is consistent with licensed chronic spontaneous urticaria dosing, which is not determined by baseline IgE or body weight [38]. Therefore, the observed association between IgE strata and UAS7 should not be interpreted as evidence that patients with higher IgE require a higher omalizumab dose. Omalizumab reduces free IgE and FcεRI-mediated activation [31,32], while studies in chronic spontaneous urticaria have linked baseline IgE and its change to treatment response [62,63]. In acute disease, IgE stratification may help identify patients with a greater allergic immune burden, although a single measurement is insufficient for individual prediction. The present data also do not establish whether 150, 300, and 450 mg differ in their speed or magnitude of clinical response. Dose selection was nonrandom and may have been influenced by disease severity and other clinical factors, creating substantial confounding by indication. Accordingly, the 450 mg exposure observed in this cohort should not be interpreted as a validated dose-escalation regimen for acute urticaria. Prospective studies should use prespecified dose groups and serially evaluate free IgE, basophil counts, FcεRI expression, basophil activation, and clinical symptom trajectories to determine whether these biomarkers can support treatment monitoring or dose selection [37].

Evidence for omalizumab has mainly come from chronic spontaneous urticaria. Randomized studies demonstrated reduced pruritus and wheal burden with good tolerability [28,30], and the X-ACT study showed benefit in patients with angioedema [64]. These data are consistent with the faster angioedema resolution observed here, but the settings differ. Chronic studies emphasize control over weeks or months, whereas this study assessed improvement within days, corticosteroid tapering, and early recurrence. The Day 1 advantage suggests that anti-IgE therapy may have value as selective early intensification in high-risk acute disease.

Treatment response also appeared to involve pathways beyond IgE. WBC, NEU, and EOS differed at admission, whereas CRP and ESR did not. Correlation and clustering analyses suggested an interconnected network involving IgE, UAS7, CRP, WBC, NEU, and EOS. Previous studies likewise indicate that multiple immune markers contribute to urticaria activity and treatment response [65], while neutrophil-to-lymphocyte and platelet-to-lymphocyte ratios may reflect inflammatory burden [66]. In this study, elevated NEU was associated with poorer short-term control, suggesting that innate inflammatory activity may complement IgE-related information when explaining heterogeneous treatment responses.

However, the predictive value of systemic inflammatory indices for treatment response remains uncertain. Tarkowski et al. [67] did not confirm the usefulness of the systemic immune–inflammation index (SII), systemic inflammation response index (SIRI), neutrophil-to-lymphocyte ratio (NLR), or platelet-to-lymphocyte ratio (PLR) as predictors of the response to omalizumab in patients with chronic spontaneous urticaria. A subsequent review by Pedersen et al. [68] also indicated that, although several candidate biomarkers for monitoring omalizumab response have been proposed, none has yet been sufficiently validated for routine individualized clinical use. Similarly, Branicka et al. [69] found that the eosinophil-, neutrophil-, platelet-, and basophil-to-lymphocyte ratios did not reliably predict the response to omalizumab in chronic spontaneous urticaria. The apparent discrepancy between these studies and our findings may partly reflect differences in disease phenotype and clinical setting. Our cohort consisted specifically of hospitalized patients with acute urticaria and substantial short-term disease burden, whereas the cited studies focused primarily on chronic urticaria during longer-term omalizumab treatment. Therefore, elevated NEU in our cohort may represent intensified innate immune activation in severe acute disease rather than a universally applicable predictor across all urticaria populations. Mechanistically, IgE may also interact with neutrophil-dependent inflammation. An experimental study demonstrated that high concentrations of IgE induced the release of neutrophil extracellular traps, with a stronger response in allergic participants [70]. This finding provides a potential biological context for the association observed in our study; however, it does not establish a causal IgE–neutrophil relationship in acute urticaria, and further mechanistic and prospective validation is required.

The corticosteroid-sparing effect was clinically important. Combination therapy reduced the total methylprednisolone-equivalent exposure and shortened tapering while overall adverse events, gastrointestinal discomfort, and hyperglycemia were less frequent. Even short glucocorticoid courses have been associated with infection, thrombosis, fractures, endocrine effects, and glucose metabolism abnormalities [26,28,30]. The improved safety profile, therefore, likely reflects both omalizumab tolerability and reduced corticosteroid exposure. This may be especially relevant for patients with diabetes, hypertension, gastrointestinal risk, or sleep and anxiety disorders.

The exploratory prediction model integrated treatment exposure and inflammatory variables. Combination therapy was associated with better outcomes, whereas elevated NEU indicated a greater risk of poor control; the model achieved an AUC of 0.812 with good calibration. As emphasized by TRIPOD and calibration research, clinical usefulness depends on both discrimination and agreement between predicted and observed risk [54,57]. Although the model is not ready for routine use, it provides a framework for identifying patients who may benefit from earlier combined therapy, including those with high UAS7, elevated IgE strata, increased NEU, angioedema, recurrence risk, or difficulty tapering corticosteroids.

Accordingly, omalizumab plus systemic glucocorticoids should not be used routinely for all cases of acute urticaria. It may instead be considered for severe disease with angioedema, slow resolution, corticosteroid dependence, early recurrence, or high risk of corticosteroid-related toxicity. This selective approach may improve control while avoiding overtreatment. However, the same clinical features that may prompt clinicians to add omalizumab can also affect subsequent outcomes, creating confounding by indication. Patients with more severe or rapidly progressive disease, angioedema, or an inadequate response to conventional therapy may have been more likely to receive omalizumab and may also have had a poorer underlying prognosis, potentially biasing the observed association against combination therapy. Conversely, unrecorded factors such as corticosteroid contraindications or intolerance, clinician preference, patient willingness, affordability, insurance coverage, and drug availability may have influenced treatment selection and could have produced bias in the opposite direction. Because these determinants were not consistently captured, the direction and magnitude of the residual indication bias cannot be determined.

The retrospective design cannot eliminate treatment-selection bias or unmeasured confounding. More specifically, omalizumab initiation was clinician-driven rather than protocolized, resulting in potential confounding by indication that is distinct from the general limitations of retrospective data. Although PSM and multivariable analyses adjusted for recorded demographic, clinical, and laboratory covariates, they could not adjust for the undocumented reasons underlying treatment selection. Consequently, the reported differences should be interpreted as adjusted associations rather than causal treatment effects. The modest sample size and the small, uneven, nonrandomly selected omalizumab dose groups prevented a reliable dose–response analysis and limited subgroup analyses and model stability. Follow-up was too short to assess chronic progression. FcεRI expression, basophil activation, and cytokines such as IL-4, IL-5, IL-13, and TSLP were unavailable, so mechanistic interpretations remain indirect. Serial post-treatment measurements of total or free IgE and basophil-related biomarkers were also unavailable. Therefore, their value for monitoring biological and clinical responses to different omalizumab doses could not be assessed. Prospective multicenter studies with longer follow-up, detailed immunological profiling, external model validation, prespecified dose groups, and repeated biomarker measurements are needed to define the optimal population, timing, and dose of omalizumab in acute urticaria. In addition, numerous secondary efficacy, safety, laboratory, subgroup, correlation, and model comparisons were conducted without formal adjustment for multiple testing. Consequently, some nominal *p*-values below 0.05, particularly those close to the significance threshold, may represent chance findings. These results should therefore be interpreted as exploratory and hypothesis-generating rather than confirmatory [59].

## 5. Conclusions

Based on real-world data from 73 hospitalized patients with acute urticaria, omalizumab combined with systemic glucocorticoids was associated with earlier reductions in UAS7 and itch VAS than systemic glucocorticoids alone, significantly shorter times to resolution of wheals and angioedema, a higher complete remission rate within 7 days (the primary efficacy outcome), and a lower recurrence rate within 14 days (the key secondary efficacy outcome). Given the possibility of residual confounding by indication, these findings represent observational associations and require confirmation in prospective studies with standardized treatment-allocation criteria. The benefits of combination therapy extended across symptom relief, resolution of skin lesions and edema, and short-term stability, suggesting that it may favorably influence the early course of severe acute urticaria.

Combination therapy also reduced total methylprednisolone-equivalent dose and shortened corticosteroid tapering time, while lowering the incidence of overall adverse events, gastrointestinal discomfort, and elevated blood glucose. IgE stratification, granulocyte-related inflammatory markers, correlation heatmaps, cluster analysis, and multivariable modeling suggest that these benefits may arise from combined suppression of free-IgE/FcεRI-related upstream activation, glucocorticoid-mediated control of inflammatory amplification, and reduced corticosteroid exposure as symptoms improve. Elevated NEU was associated with a greater risk of poor control, whereas combination therapy was associated with better short-term outcomes. The AUC of 0.812 and good calibration indicate that a model combining immune–inflammatory markers and treatment-exposure variables may have value for risk stratification.

Overall, omalizumab combined with systemic glucocorticoids may represent an optimized treatment option for patients with severe acute urticaria accompanied by angioedema, high short-term recurrence risk, or risk of glucocorticoid-related adverse effects. However, this strategy remains off-label and should not be considered routine treatment until its efficacy, safety, cost-effectiveness, and appropriate patient-selection criteria have been confirmed in prospective studies.

## Figures and Tables

**Table 1:** Demographic, clinical, and laboratory characteristics of the study population.

Characteristics	Overall Cohort (*n* = 73)	Corticosteroid Group (*n* = 39)	Combination Therapy Group (*n* = 34)	*p*-Value
Age (years), median (IQR); range	36.0 (22.0–52.0); 8–86	36.0 (23.0–52.0); 8–78	37.5 (19.0–54.0); 11–86	0.423
Age category (%)	Pediatric: 11 (15.1); adult: 62 (84.9)	Pediatric: 4 (10.3); adult: 35 (89.7)	Pediatric: 7 (20.6); adult: 27 (79.4)	0.327
Sex (%)	Female: 42 (57.5); male: 31 (42.5)	Female: 27 (69.2); male: 12 (30.8)	Female: 15 (44.1); male: 19 (55.9)	0.012
Body weight (kg), median (IQR)	65.0 (57.7–70.3), *n* = 72	65.0 (58.0–70.0), *n* = 38	65.0 (56.0–72.0), *n* = 34	0.288
BMI (kg/m^2^), median (IQR)	27.8 (25.1–29.5)	27.3 (24.2–29.3)	28.0 (26.1–29.7)	0.312
Length of hospital stay, days, median (IQR)	7.0 (6.0–9.0)	7.0 (6.0–9.0)	7.5 (6.0–9.0)	0.237
Identifiable precipitating factor (%)	Yes: 52 (71.2); No: 21 (28.8)	Yes: 28 (71.8); No: 11 (28.2)	Yes: 24 (70.6); No: 10 (29.4)	0.812
Alcohol consumption history (%)	Yes: 17/71 (23.9); No: 54/71 (76.1)	Yes: 9/39 (23.1); No: 30/39 (76.9)	Yes: 8/32 (25.0); No: 24/32 (75.0)	0.612
Comorbidities (%)	Hypertension: 14 (19.2); diabetes: 8 (11.0); cardiovascular disease: 7 (9.6); other: 4 (5.5); none: 40 (54.8)	Hypertension: 8 (20.5); diabetes: 5 (12.8); cardiovascular disease: 4 (10.3); other: 2 (5.1); none: 20 (51.3)	Hypertension: 6 (17.6); diabetes: 3 (8.8); cardiovascular disease: 3 (8.8); other: 2 (5.9); none: 20 (58.8)	0.964
WBC (×10^9^/L), median (IQR)	11.70 (9.27–14.62)	13.13 (9.51–15.09)	11.32 (8.37–13.59)	0.046
NEU (×10^9^/L), median (IQR)	9.39 (7.45–12.13), *n* = 72	9.56 (7.86–12.46), *n* = 39	9.28 (6.84–11.68), *n* = 33	0.032
EOS (×10^9^/L), median (IQR)	0.10 (0.00–0.20), *n* = 70	0.10 (0.05–0.25), *n* = 38	0.10 (0.00–0.20), *n* = 32	0.038
LYM (×10^9^/L), median (IQR)	7.19 (5.47–9.15)	7.14 (5.14–9.20)	7.30 (5.52–8.92)	0.187
PLT (×10^9^/L), median (IQR)	227.7 (201.1–249.9)	235.6 (206.8–255.9)	223.7 (190.8–244.0)	0.223
CRP (mg/L), median (IQR)	8.94 (3.65–29.45), *n* = 66	8.34 (2.51–31.51), *n* = 34	8.94 (5.41–23.35), *n* = 32	0.718
ESR (mm/h), median (IQR)	12.49 (4.99–22.49), *n* = 42	13.49 (5.98–23.99), *n* = 22	8.50 (4.49–20.00), *n* = 20	0.412
Total IgE (IU/mL), median (IQR)	102.0 (52.3–324.6), *n* = 59	97.0 (58.2–206.6), *n* = 30	188.0 (49.7–385.3), *n* = 29	0.197

**Table 2:** Conversion of clinical effect sizes for the primary and key secondary efficacy outcomes.

Clinical Endpoints	Control Group	Experimental Group	Absolute Difference	Relative Change	Clinical Interpretation	*p*-Value
7-Day Complete Remission Rate	53.3%	82.9%	Increase of 29.6%	RR = 1.56	Approximately one additional patient achieves complete remission within 7 days for every four patients treated.	0.002
14-Day Recurrence Rate	40.0%	17.1%	Decrease of 22.9%	57.3% relative decrease	Approximately one short-term recurrence is prevented for every five patients treated.	0.003
Time to Resolution of Urticaria	Longer	Shorter	Shorter in the combination group	Consistent direction	Indicates that combination therapy accelerates wheal resolution	<0.001
Time to Resolution of Angioedema	Longer	Shorter	Shorter in the combination group	Consistent direction	Indicates improved control of deep edema	<0.001
Early Decrease in UAS7	Slower decline after Day 1	Rapid decline after Day 1	Greater early decline in the combination group	Consistent direction	Indicates that treatment differences emerge early	Day 1: 0.021
Early Decrease in Itch VAS	Slower decline after Day 1	Rapid decline after Day 1	Greater early decline in the combination group	Consistent direction	Indicates earlier antipruritic effects	Day 1: 0.032

**Table 3:** Clinical effect measures for safety endpoints.

Safety Endpoints	Control Group	Experimental Group	Absolute Difference	Relative Change	Clinical Interpretation	*p*-Value
Overall Adverse Reactions	46.7%	26.8%	Decrease of 19.9%	42.6% relative decrease	Approximately one adverse event is prevented for every six patients treated.	0.019
Gastrointestinal Discomfort	17.7%	9.8%	Decrease of 7.9%	44.6% relative decrease	Suggests reduced corticosteroid-related gastrointestinal burden	0.048
Elevated Blood Glucose	13.2%	4.9%	Decrease of 8.3%	62.9% relative decrease	May benefit patients at risk of glucose metabolism abnormalities	0.038
Insomnia and Anxiety	19.1%	9.8%	Decrease of 9.3%	48.7% relative decrease	Downward trend; requires confirmation in a larger sample	0.077
Blood Pressure Fluctuations	12.0%	4.9%	Decrease of 7.1%	59.2% relative decrease	Downward trend; requires confirmation in a larger sample	0.058
Serious Adverse Events	10.3%	2.9%	Decrease of 7.4%	71.8% relative decrease	Suggests that combination therapy did not increase serious safety risk	0.031

**Table 4:** Interpretation of key variables in a multifactor logistic model.

Variables	OR	95%CI	*p*-Value	Interpretation of Results
IgE levels	1.32	0.98–1.78	0.071	Higher immune burden may influence short-term control
Hormone dosage	0.62	0.45–0.85	0.003	Higher exposure is associated with outcomes but should be interpreted alongside disease severity
CRP	1.21	0.88–1.66	0.247	Conventional systemic inflammatory marker with limited explanatory value
NEU	1.41	1.02–1.95	0.038	Neutrophilic inflammatory burden is an independent risk marker
EOS	1.18	0.81–1.72	0.382	No independent predictive role was demonstrated
Combination therapy	0.47	0.28–0.79	0.004	Combination therapy is associated with a lower risk of unfavorable outcomes

**Table 5:** Summary of overall prediction model performance.

Model Metrics	Results	Clinical Implications
AUC	0.812	Good discrimination; may support preliminary risk stratification
95% CI	0.726–0.898	Discrimination was relatively stable within the internal sample
Brier score	0.126	Predicted probabilities were close to observed outcomes
Calibration curve	Approaching the ideal curve	No substantial systematic over- or underestimation was observed

## Data Availability

All data generated or analyzed during this study are included in this published article. Further information is available from the corresponding author on reasonable request.
